# Supplementary material for: Biologic Impact of Green Synthetized Magnetic Iron Oxide Nanoparticles on Two Different Lung Tumorigenic Monolayers and a 3D Normal Bronchial Model—EpiAirwayTM Microtissue
Source: Pharmaceutics. 2022 Dec 20;15(1):2. doi: 10.3390/pharmaceutics15010002 (PMC9866254; doi:10.3390/pharmaceutics15010002)
Supplement: Supplementary file 1 [file pharmaceutics-15-00002-s001.zip › pharmaceutics-2068286-supplementary.pdf]

pharmaceutics

Supplementary Material

Biologic impact of green magnetic nanoparticles on two different lung tumorigenic monolayers and a 3D normal bronchial model—EpiAirway™ microtissue

Elena-Alina Moacă [1,2], Claudia Watz [1,2,*], Alexandra Faur [3], Daniela Lazăr [3], Vlad Socoliuc [4,5], Cornelia Păcurariu [6], Robert Ianoș [6], Cristiana-Iulia Rus [6], Daliana Minda [1,2], Lucian Barbu-Tudoran [7,8] and Cristina Adriana Dehelean [1,2]

S1. Materials and Methods

S1.1. In vitro evaluations

S1.1.1. Culture Procedure

The specific cell culture media used for proliferation of A549 cells was Dulbecco's modified Eagle's medium (DMEM, code 30-2002) enriched with 10% fetal bovine serum (FCS, code ATCC 30-2020) and 1% antibiotics mixture of penicillin and streptomycin, whereas the culture media used for culturing the NCI-H460 cells were a RPMI-1640 medium (code ATCC 30-2001) containing 10% FCS and 1% penicillin/streptomycin mixture.

All in vitro experiments were conducted under sterile conditions by using the MSC Advantage 12 model biosafety cabinet (Thermo Fisher Scientific, Inc., Waltham, MA, USA) and an incubator (Steri-Cycle i160 model from Thermo Fisher Scientific, Inc., Waltham, MA, USA) with humidified atmosphere and 5% CO_2.

S1.1.2. Cell viability assessment by means of the Alamar blue colorimetric test

To quantify the effect induced by test samples (Cs 25, Cs 80 and Ob 25, Ob 80) at different concentrations (150, 300, 500 µg/mL) and 4 time intervals (24, 48, 72, 96 h) on two different lung cancer cell lines (A549 and NCI-H460), the Alamar blue colorimetric assay was performed. In brief, both cell lines were plated in 96-well plates to a density of $1x10^4$ cells/well and were maintained in humidified atmosphere at 37 °C and 5% CO_2 until the 80% confluence was reached. Afterwards, the cell medium was removed and the cell monolayer was treated with test samples at concentrations of 150, 300, 500 µg/mL for 24 h, 48 h, 72 h, and 96 h.

Control cells were maintained under the same conditions as the sample-treated ones; however, they were exposed only to the specific culture medium.

S1.1.3. Cytotoxicity assay by quantifying the lactate dehydrogenase (LDH) released

To evaluate the cytotoxic potential of test samples (Cs 25, Cs 80 and Ob 25, Ob 80) at different concentrations (150, 300, 500 µg/mL) on A549 and NCI-H460, the lactate dehydrogenase (LDH) release assay was performed. The protocol employed for this method is closed to the one described for the Alamar blue test. Nevertheless, at the end of the stimulation interval, an amount of 50 µL/well media (containing the extracellular LDH leakage) was transferred into another 96-well plate and mixed with 50 µL/well reaction mixture several times. Afterwards, the plate was stored in a dark chamber for 30 min and, in the end, the reaction was stopped by adding 50 µL/well stop solution provided in the LDH kit (LDH Cytotoxicity Assay, Code no C20301, Thermo Scientific LSG).

S1.1.4. Biosafety profile using the EpiAirway™ 3D in vitro microtissues through MTT test

To determine the biosafety level of test samples (Cs 25, Cs 80 and Ob 25, Ob 80) at the highest two test concentrations (300, 500 µg/mL), the EpiAirway™ model was employed, followed by the MTT test. In brief, upon arrival, the inserts were carefully removed from agarose and transferred into 6-well plates prefilled with 1 mL/well of assay medium (AIR-100-ASY), afterwards the tissues were incubated overnight under standard conditions (37 °C, 5% CO_2). At the end of the equilibration period, the tissues are ready for the experiments, so the old medium is replaced with a newly fresh one. However, the apical surface of the tissues was rinsed twice with 400 µL TEER buffer. After that, the tissue inserts were treated with 50 µL of test samples for 24h.

Before performing the MTT assay for quantifying the EpiAirway™ tissue viability, each insert was washed for three times with 400 µL of TEER buffer. The next step consisted in placing the inserts into a 24-well plate pre-filled with 300 µL of MTT reagent for 90 min and just before the end of the incubation time, 2 mL of MTT extractant solution was added into the wells.

S2. Results

S2.1. Cell morphology assessment

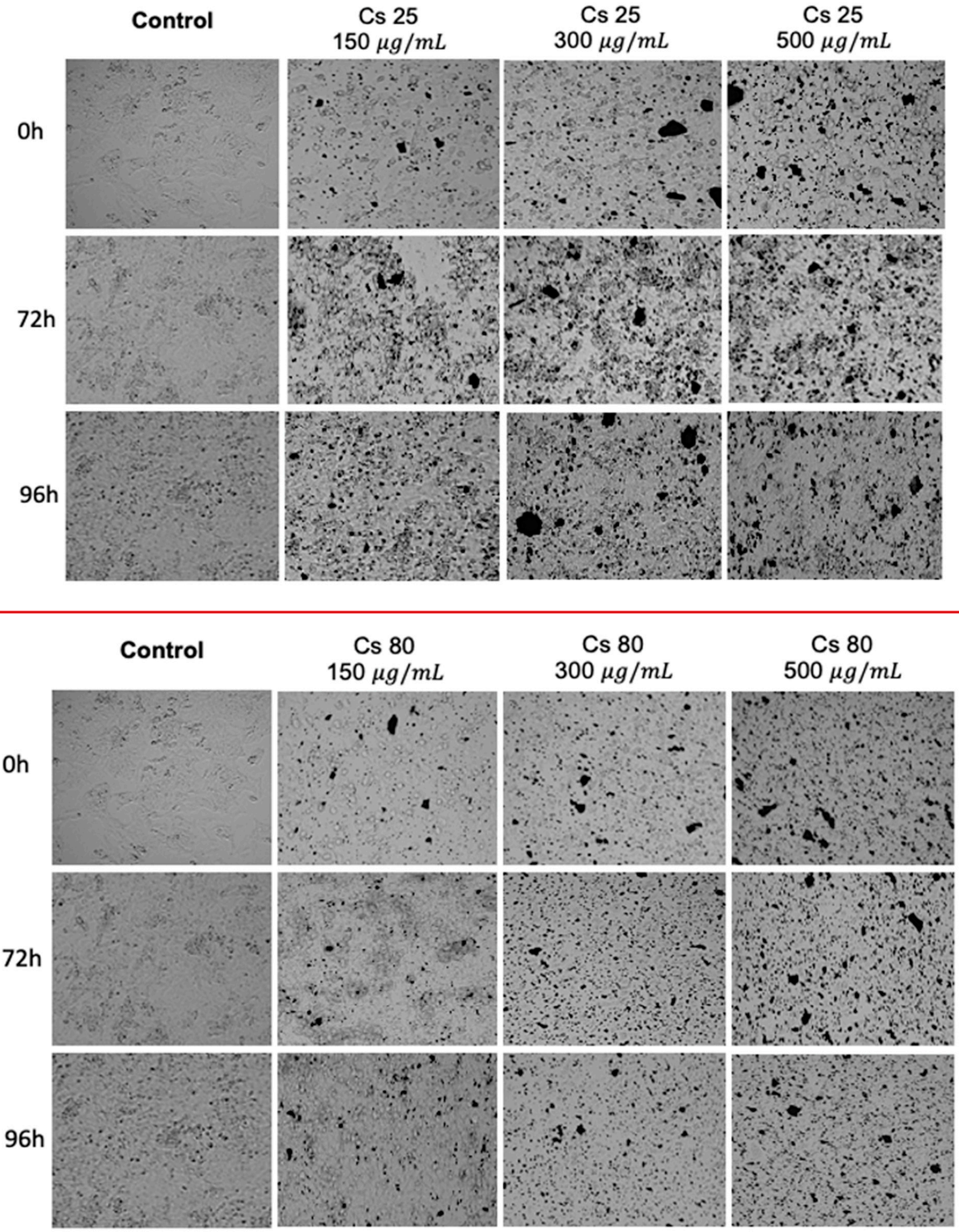

Figure S1. Cell morphology of A549 cells treated with Cs 25 and Cs 80 (concentration of 150, 300, 500 μg/mL) at initial time (0 h) and 72–96 h post-exposure.

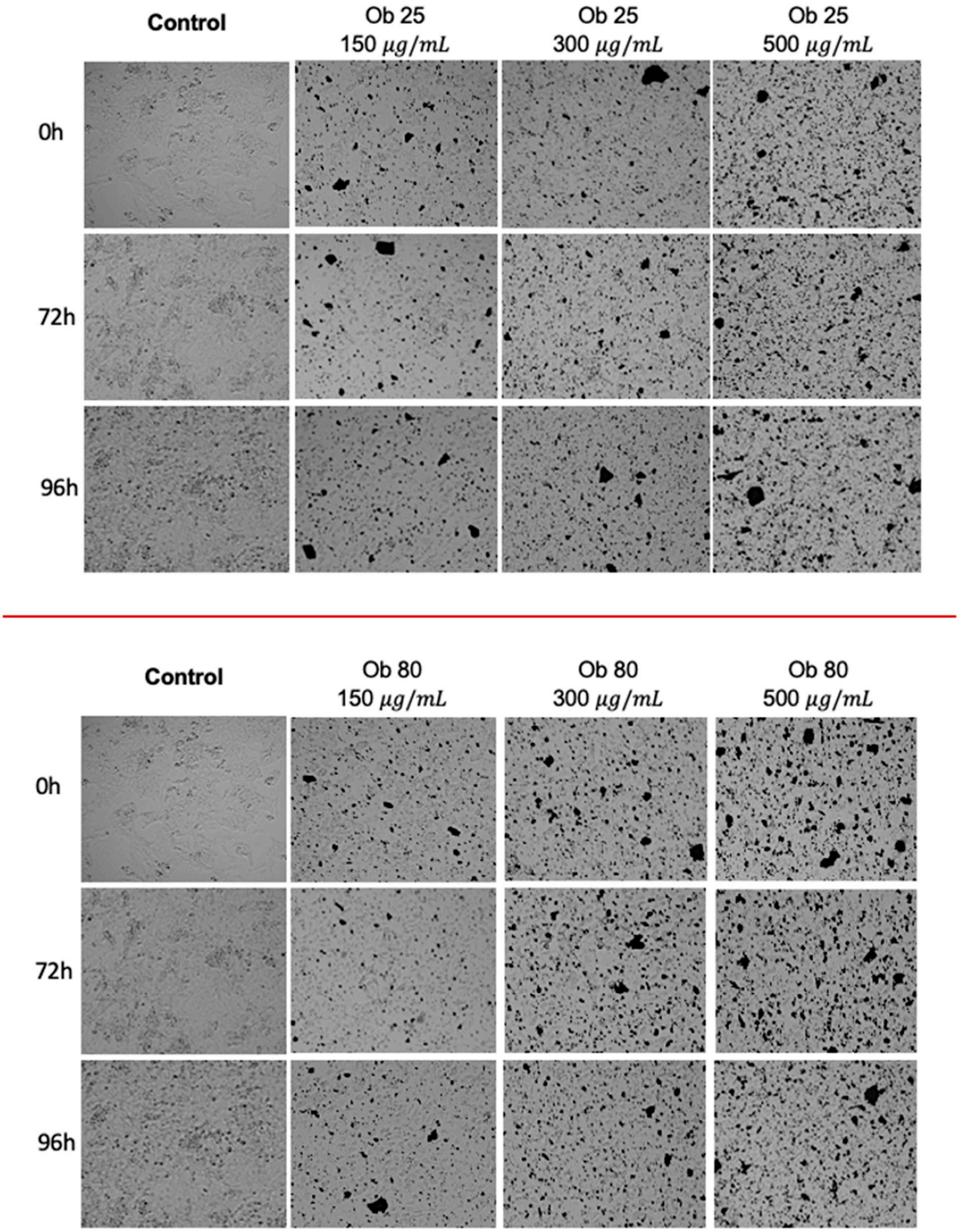

Figure S2. Cell morphology of A549 cells treated with Ob 25 and Ob 80 (concentration of 150, 300, 500 μg/mL) at initial time (0 h) and 72–96 h post-exposure.

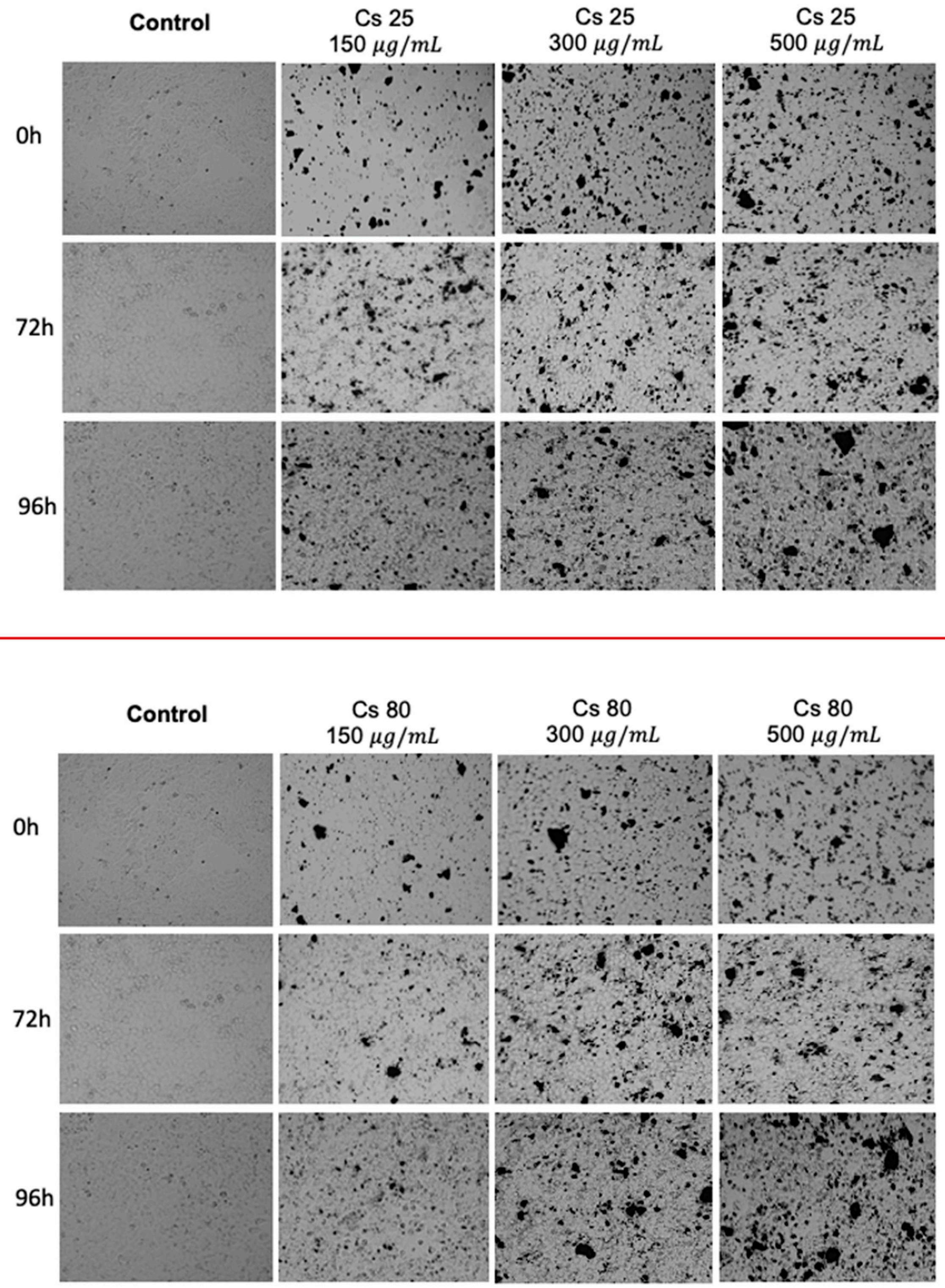

Figure S3. Cell morphology of NCI-H460 cells treated with Cs 25 and Cs 80 (concentration of 150, 300, 500 μg/mL) at initial time (0 h) and 72–96 h post-exposure.

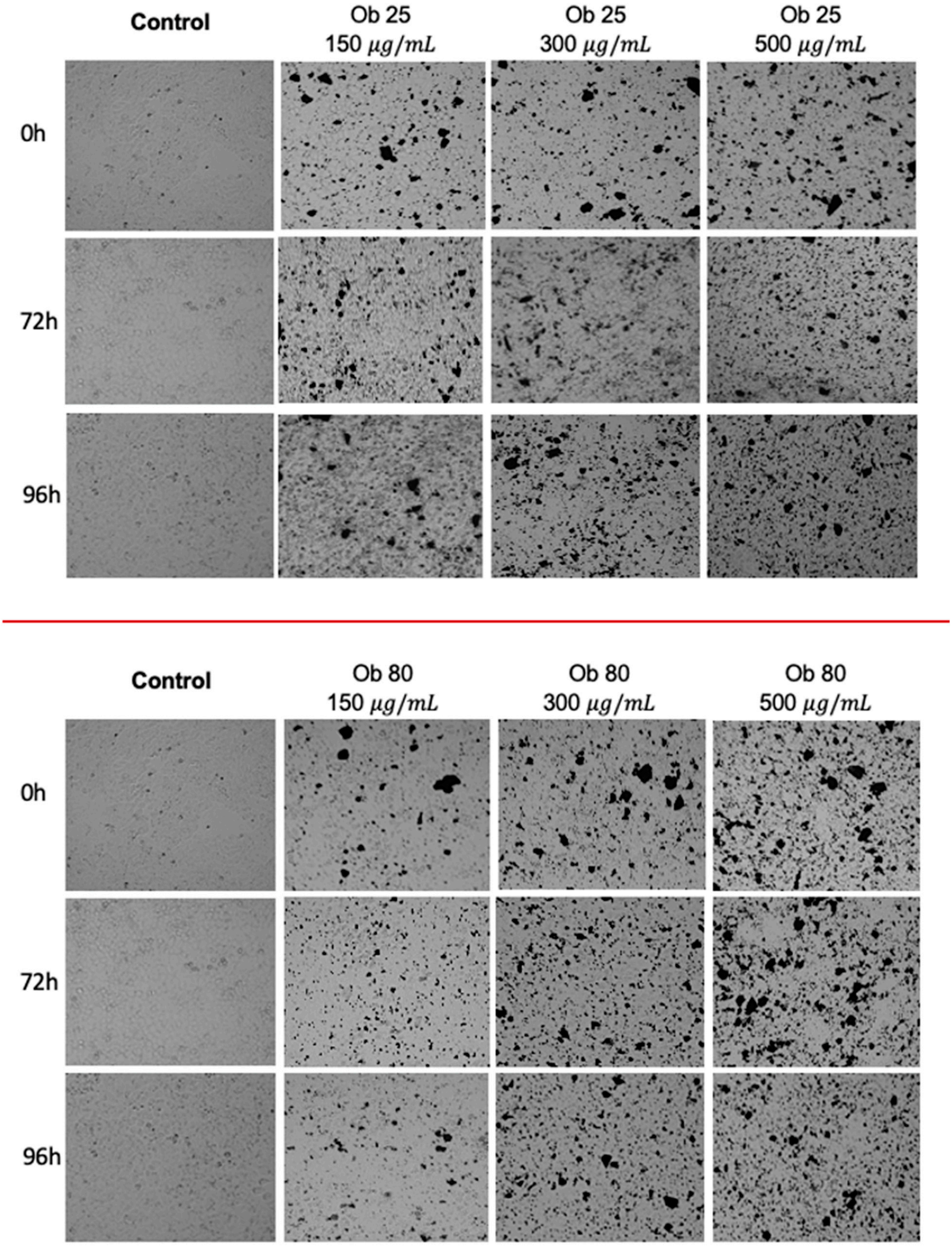

Figure S4. Cell morphology of NCI-H460 cells treated with Ob 25 and Ob 80 (concentration of 150, 300, 500 μg/mL) at initial time (0 h) and 72–96 h post-exposure.
